# Microcapsule Triggering Mechanics in Cementitious Materials: A Modelling and Machine Learning Approach

**DOI:** 10.3390/ma17030764

**Published:** 2024-02-05

**Authors:** Evan John Ricketts, Lívia Ribeiro de Souza, Brubeck Lee Freeman, Anthony Jefferson, Abir Al-Tabbaa

**Affiliations:** 1 School of Engineering, Cardiff University, 3-5 The Walk, Cardiff CF24 3AA, UK or brubeck.freeman@lusas.com (B.L.F.); jeffersonad@cardiff.ac.uk (A.J.); 2 Department of Engineering, University of Cambridge, Trumpington Street, Cambridge CB2 1PZ, UK; livia@mimicrete.com (L.R.d.S.); aa22@eng.cam.ac.uk (A.A.-T.); 3 LUSAS, Forge House, 66 High Street, Kingston upon Thames KT1 1HN, UK

**Keywords:** self-healing concrete, microcapsules, triggering mechanics, continuum damage modelling, finite element modelling, microfluidics, machine learning, neural networks, design curves, interfacial properties

## Abstract

Self-healing cementitious materials containing microcapsules filled with healing agents can autonomously seal cracks and restore structural integrity. However, optimising the microcapsule mechanical properties to survive concrete mixing whilst still rupturing at the cracked interface to release the healing agent remains challenging. This study develops an integrated numerical modelling and machine learning approach for tailoring acrylate-based microcapsules for triggering within cementitious matrices. Microfluidics is first utilised to produce microcapsules with systematically varied shell thickness, strength, and cement compatibility. The capsules are characterised and simulated using a continuum damage mechanics model that is able to simulate cracking. A parametric study investigates the key microcapsule and interfacial properties governing shell rupture versus matrix failure. The simulation results are used to train an artificial neural network to rapidly predict the triggering behaviour based on capsule properties. The machine learning model produces design curves relating the microcapsule strength, toughness, and interfacial bond to its propensity for fracture. By combining advanced simulations and data science, the framework connects tailored microcapsule properties to their intended performance in complex cementitious environments for more robust self-healing concrete systems.

## 1. Introduction

Self-healing technologies aim to impart materials, such as polymers and cementitious composites, with the ability to autonomously repair damage. Microcapsules are a critical component of many self-healing systems, enabling the incorporation and release of healing agents into structural materials to seal cracks [1,2,3]. Different healing agents have been encapsulated, including polymers, bacteria, and chemicals, such as sodium silicate and dicyclopentadiene, that can react with the host material or each other to re-bond crack faces [2,3,4]. Microcapsules for self-healing applications are commonly fabricated using emulsification techniques such as microfluidics [5], complex coacervation [6], in situ polymerisation [3], and interfacial polymerisation [6]. Each technique presents challenges in precisely controlling microcapsule size, morphology, shell thickness and properties. Tailoring the mechanical properties and geometry of the microcapsule shell through techniques like microfluidics and interfacial polymerisation remains challenging yet critical for optimising the rupture and targeted release of healing agents into cracks in concrete. Quantifying the shell’s mechanical properties and adhesion to the cementitious matrix is difficult, but this is needed to relate shell properties to rupture triggering [7,8]. Optimising microcapsule content in concrete is important to balance healing efficiency and the impact of the capsules on matrix properties. Advanced characterisation techniques are required to fully relate shell properties and cementitious matrix factors to microcapsule activation, rupture, and healing agent release for enhanced self-healing system design [9].

To date, much of the work on modelling microcapsule-based self-healing cementitious materials (SHCMs) has focussed on the calculation of the effective properties of the composite using micromechanical models [7] and homogenisation techniques [10]. Further applications of modelling to such materials have included predicting the probability of a crack hitting a microcapsule [11], mechanical self-healing [12], investigations of fracture mechanisms [13], and the survivability of microcapsules during the mixing process [14]. The investigation of fracture mechanisms can aid the design and development of SHCMs utilising microcapsules through the determination of the mechanical properties of the shell and interfacial bond required for rupture. In Ponnusami et al. (2015) [15] (see also [16,17]), a cohesive zone model was used to investigate the fracture of micro-particles in a matrix material. The authors explored the effect of varying a range of mechanical properties, interfacial bond and the effect of existing flaws. The results were used to produce fracture maps that indicate whether a particle is expected to fracture for given properties relative to the matrix material. A similar approach was taken by Gilabert et al. (2017) [18], who employed extended finite elements to investigate capsule rupture in capsule-based self-healing polymers with varying mechanical properties in addition to the homogenised properties of the capsule–polymer matrix composite. Reda and Chidiac (2022) presented a numerical investigation into the rupture and debonding of microcapsules in a mortar matrix [9]. The study investigated the effects of microcapsule mechanical properties and geometry, as well as the effect on these properties of the age of the mortar and the use of supplementary cementitious materials in the mix design. The authors found that the mode of failure (i.e., rupture vs. debonding) was significantly affected by the age of the mortar and that rupture was dominant for 66% of capsules embedded in 28-day-old mortar specimens.

The use of numerical models to undertake such investigations can be considered to be a ‘virtual’ testing approach. Virtual testing can provide valuable insights into the physical processes and mechanical response of SHCMs [19]. In addition, virtual testing has the benefit of allowing for the investigation of a large parameter space that would be impracticable using physical tests [20]. In the present work, a continuum damage mechanics model [21] is employed for the virtual testing of microcapsule fracture under varying mechanical properties of the microcapsule itself and the interfacial bond with the cementitious matrix. The data generated from the virtual testing are then fed into a machine learning model, which acts as a surrogate model that can give almost instantaneous predictions of microcapsule fracture.

Machine learning has most frequently been applied to problems of classification and regression, whereby a discrete categorical value or numerical value is predicted, respectively [22]. Here, our problem is that of classification, where the model will determine whether there is fracture or non-fracture of an embedded microcapsule. Various machine learning methods such as decision trees and logistic regression can be applied for classification problems, but often artificial neural networks (ANNs) are employed due to their ability to capture the overall behaviour when tuned appropriately. Currently, ANNs have been widely applied in civil engineering for cementitious materials in areas such as concrete mix design or material property estimation for modulus of elasticity [23], compressive and tensile strength [24,25,26], and dry shrinkage [27]. See Kekez and Kubica (2021) [28] for an excellent summary of many other machine learning applications in relation to cementitious materials.

Whilst there have been many wider applications, machine learning has had much less application in SHCMs [29,30,31,32,33,34]. This is especially true when considering the application of machine learning to cementitious materials containing microcapsules. Marani and Nehdi (2020) [35] compared the use of different ML methods, a combination of random forest and gradient boosting regression, to predict the compressive strength of phase change material-incorporated cement composites. All of these methods provide relatively accurate predictions, with R^2^ values between 0.93 and 0.97. Parameter importance analysis showed that in all cases, fine aggregate content, microcapsule content, and age were the most influential when training, which is consistent with the experimental findings in the literature. It was concluded that larger datasets are needed to be able to determine the importance of the input into such ML models more clearly and that their application results in a deeper understanding of the experimental data, leading to more robust predictive tools.

Microcapsules are a critical component that enable the incorporation and release of healing agents in self-healing cementitious systems. However, their success depends on tailoring the shell properties to survive initial mixing while still rupturing upon matrix cracking to release the healing agent. This requires an understanding of the mechanics governing microcapsule triggering and compatibility with the cementitious matrix. The complex, heterogeneous, and evolving nature of concrete adds further challenges. This study aims to relate microcapsule properties to their triggering behaviour through an integrated modelling and machine learning approach. Microfluidics is first utilised to produce acrylate-based microcapsules with adjustable shell properties. These are experimentally characterised and then virtually tested in cementitious matrices using a model based on continuum damage mechanics. A large parametric study is performed to generate data for training a machine learning model to rapidly predict microcapsule rupture. The modelling and data science framework seeks to provide an efficient pathway to design microcapsules tailored for triggering within cementitious materials. This could enhance the robustness of self-healing concrete by reducing the need for extensive physical testing.

## 2. Materials and Methods

To produce the double emulsion, a microfluidic device with four parallel flow focusing channels was used in a Telos device (Dolomite Microfluidics, Royston, UK), as described elsewhere [5]. Double emulsions with an organic core were created using mineral oil (light, Sigma Aldrich, St. Louis, MO, USA). As a precursor for the shell in the middle phase, ethylene glycol ether phenyl acrylate (PEA, Sigma Aldrich) containing the crosslinker 1,6-hexanediol diacrylate (HDDA, Sigma Aldrich) in the range of 0–50 wt% and 1 wt% of hydroxy-2-methylpropiophenone photoinitiator was used. For the outer/continuous phase, an aqueous solution with 5% polyvinyl alcohol (MW 13,000–23,000, 87–89% hydrolysed) is employed. Middle and outer fluids were injected using pressure pumps (Dolomite Microfluidics, UK) at typical flow rates of 93 μL/min^−1^ and 400 μL/min^−1^, respectively. Inner fluids were injected with syringe pumps at flow rates of 80 μL/min^−1^. The shell was polymerised using a UV lamp (Omnicure, St. Louis, MO, USA, 50% aperture) exposed over the collection tube shortly after the formation of double emulsion droplets. To prevent microcapsule aggregation during polymerisation, the microcapsules were collected in an aqueous solution containing 5% by weight of PVA. Using an optical microscope (DM 2700 M, Leica, Wetzlarm, Germany), the outer and inner diameters of the manufactured double emulsions and microcapsules were determined. To investigate the behaviour of microcapsules in cement, ordinary Portland cement (CEM I 42.5) supplied by Heidelberg-UK and water (w/c = 0.45) were combined with microcapsules containing mineral oil as the core and polymerised PEA as the shell. The mixture was then poured into oiled silicone moulds measuring 10 × 10 × 50 mm^3^. After 28 days of curing, the samples were broken, allowing for the observation of oil leaking from the microcapsules into the sample. A Zeiss Evo LS15 scanning electron microscope (SEM) (Jena, Germany) was utilised to qualitatively examine the interfacial bond between the capsule and the cement.

To quantitatively evaluate the mechanical properties of each polymer, the mixtures were polymerised into ASTM Type D638 [36] dogbone specimens for tensile testing. Specifically, negative moulds were cast in poly(dimethylsiloxane) (PDMS). A volume of 1.5 mL of the prepared liquid polymer was then injected into the PDMS mould, covered with a glass slide to prevent meniscus formation, and polymerised under light for 4 min. Uniaxial tensile tests were carried out using an Instron Testing System at a constant pulling rate of 4 mm/min. The measured force was normalised by the undeformed cross-sectional area to obtain stress, whilst the extension was normalised by the initial gauge length to obtain strain. The loading slope was used to determine Young’s modulus. The maximum stress and strain values prior to failure were defined as the ultimate tensile strength and failure strain, respectively.

Here, we apply machine learning techniques to determine the fracture behaviour of microcapsules through classification. Explicitly, we want to determine whether a set microcapsule will fracture based on capsule and interfacial parameters. Initial trials were conducted using random forests for classification, but it was observed that whilst the model had elevated levels of accuracy when cross-validating, the overall microcapsule fracture behaviour was not captured, indicating signs of overfitting. Whilst this could have been mitigated through parameter tuning, it is entirely possible that this method would be unsuitable for the given dataset. Thus, artificial neural networks were employed. The dataset used for training contained 20,412 data points and was generated using the finite element model described in Section 3.3. A summary of the inputs and outputs of the network is given in Table 1).

## 3. Results and Discussion

### 3.1. Characterisation of the Shell

**Figure 1:**
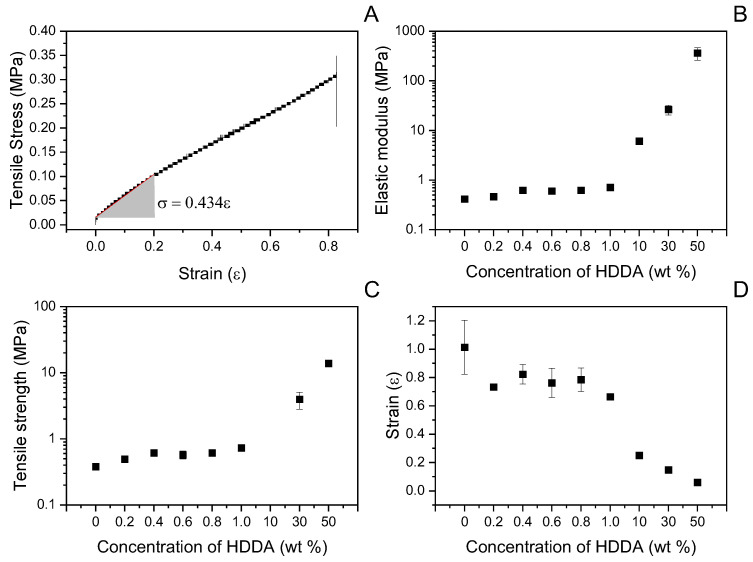
Mechanical properties of acrylate with a range of crosslinker concentrations. (**A**) Typical stress–strain response from an acrylate hydrogel sample. The slope of the linear section of the response was used to extract the elastic (Young’s) modulus of the specimen; (**B**) the Young’s modulus; (**C**) failure stress; and (**D**) failure strain.

**Figure 2:**
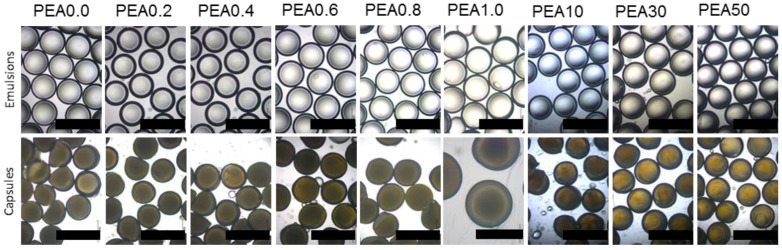
Controlled formation of microcapsules using microfluidics with different mechanical properties. (**top row**) Optical micrographs showing W/O/W double emulsions. (**bottom row**). Scale bars are 1000 μm, except for optical micrographs of PEA 1.0, where the scale bar is 500 μm. The numbers in the labels denote the concentration of crosslinker.

In this study, we aimed to develop acrylate materials with adjustable mechanical properties that could potentially be used in self-healing concrete shells [5,37]. We utilised ethylene glycol phenyl ether acrylate (PEA) as the main monomer and 1,6-hexanediol diacrylate (HDDA) as a crosslinker at concentrations ranging from 0 to 50 wt%, as shown in Figure 1 and Figure 2. We performed mechanical testing to determine tensile strength, modulus of elasticity, and elongation at fracture. Increasing the HDDA concentration resulted in an increase in tensile strength from 0.4 to 14 MPa (Figure 1C) and modulus of elasticity from 0.4 to 363 MPa (Figure 1B), while elongation at fracture decreased with higher HDDA concentration (Figure 1D). These results demonstrate that a wide range of mechanical properties can be achieved by varying monomer composition and crosslink density. The 4-order and 3-order variations in modulus and tensile strength, respectively, allow broad parameter tuning for modelling. The lower tensile strength may be attributed to differences in chemistry and curing as compared to other studies [38]. Further increasing stiffness and strength is possible by adding IBOA, ETMPTA, or more crosslinkers. Overall, the acrylate system allows for customisable mechanics through straightforward formulation changes.

### 3.2. Production of Microcapsules and Test of Interfacial Bond

Using microfluidics, double emulsions were produced with mineral oil as the model core content, and PEA with different contents of crosslinker HDDA as the precursor of the shell and PVA 5 wt% as the outer phase at flow rates of 86, 96 and 400 μL/min for inner, middle, and outer, respectively. After the polymerisation, capsules with a size around 550 μm and a shell of 36 μm were produced. The typical production rate was around 9 g/h, which is typical for capsules produced in this microfluidic platform [5]. Images of the produced microcapsules can be seen in Figure 2.

**Figure 3:**
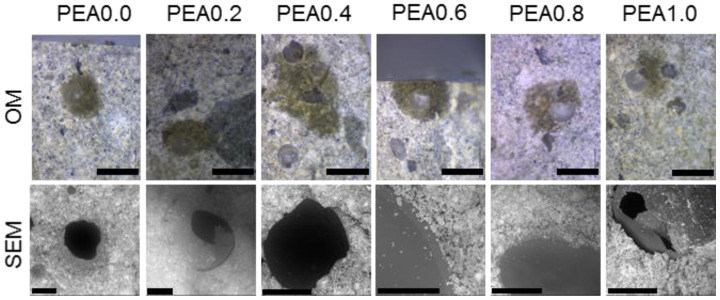
(**top row**) Optical micrographs of the microcapsules embedded in cement paste—scale bars are 1000 μm. (**bottom row**) SEM images of microcapsules in cement paste. Scale bars are 200 μm, except for PEA0.6, which represents 100 μm.

To qualitatively explore the interfacial bonding between the capsule and the cement paste, after the successful production of the capsules, the material was embedded in cement paste (w/c = 0.45). After 28 days, the prism was broken, and its surface was observed using SEM. The results were as expected: the PEA with a lower crosslinker content presented good interfacial bonding, and all the capsules were triggered. This was also observed in a recent publication, where the PEA shell presented an excellent bond with the cementitious matrix [5]. With increased crosslinker content, the interfacial bonding became poorer. For the capsule with 1 wt% HDDA, the material presented with a poor bond with the cement paste, as observed by the dark and smooth debonded shell surface with few hydration products. This indicates the crosslinker sharply increases the hydrophobicity of the shell, thus decreasing the interfacial bonding and favouring debonding. An optical micrograph and SEM images of the produced microcapsules embedded in the cement paste can be seen in Figure 3.

### 3.3. Predicting Rupture

**Figure 4:**
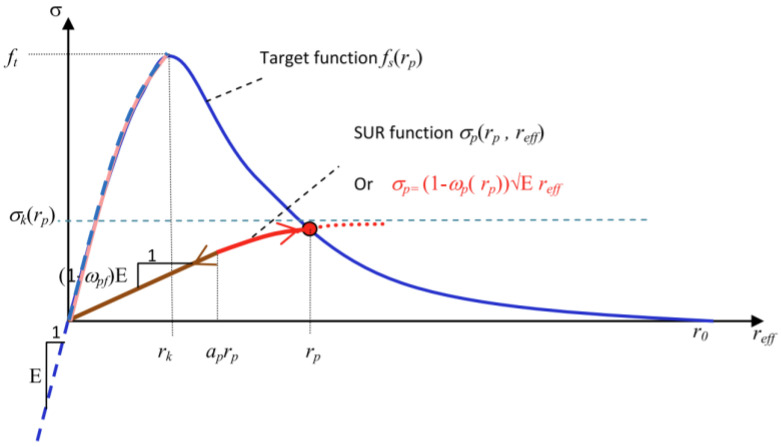
SUR function and target exponential softening curve (reproduced from Alnaas and Jefferson (2016) [21] with permission).

The triggering of microcapsules embedded in a matrix material is dependent upon the mechanical properties of the shell, as well as the interfacial bond (see Figure 3). The results of Section 3.1 and Section 3.2 show that these properties can be tuned via variations to the monomer composition and crosslink density. To tailor the microcapsule shell properties to ensure physical triggering in a range of matrix materials, an understanding of the fracture mechanisms and the effect on the fracture response of varying the mechanical properties of both the matrix material and the interfacial bond is required. In the present work, we undertake a virtual testing approach using the continuum damage mechanics (CDMs)-based finite element model presented in Alnaas and Jefferson (2016) [21]. In the model, the stress (σ) and strain (ε) tensors are related through the following equation:

(1) $$\sigma =\left(1-\omega \right)D:\varepsilon$$

in which D is the elasticity tensor and $\omega \in \left[0,1\right]$ is a scalar damage variable (where 0 indicates undamaged material and 1 indicates complete failure). Damage is assumed to initiate once the tensile stress exceeds the tensile strength of the material; whilst the evolution of the damage variable is calculated using a smooth unload–reload (SUR) function that is based on a standard exponential softening curve. A depiction of the SUR function ($\sigma_{p}$) and target exponential softening curve ($f_{s}$) can be seen in Figure 4, in which E is Young’s modulus, $\sigma_{k}$ is the target stress, $\omega_{p}$ and $\omega_{pf}$ are damage parameters and $r_{k}$, $r_{p}$, and $r_{eff}$ are damage evolution parameters. The strain at the effective end of the softening curve is calculated from the fracture energy following the crack band approach of Bažant and Oh (1983) [39].

**Figure 5:**
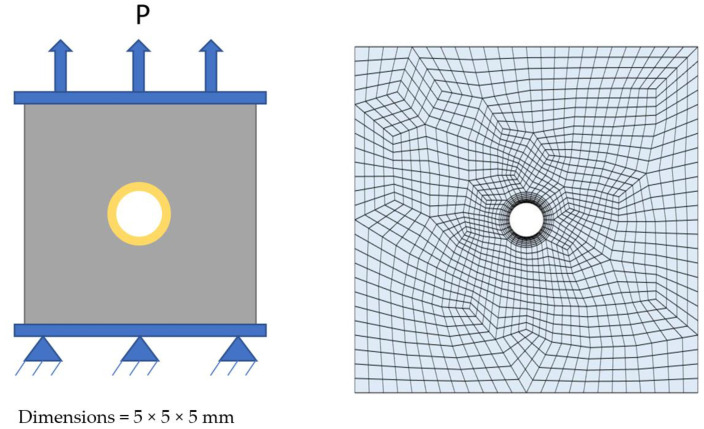
(**Left**) problem set up and (**Right**) finite element mesh.

To test for microcapsule fracture, we consider the loading of a cementitious specimen with a centrally located microcapsule in direct tension to a strain of 0.1%. The interfacial transition zone between the microcapsule and cementitious matrix was represented as a region of finite thickness of 100 μm [40]. The problem setup and a depiction of the finite element mesh used in the analyses can be seen in Figure 5. It is noted that in the analyses, the cargo material within the microcapsules was assumed not to contribute to the mechanical response. To validate the model predictions prior to the virtual testing, the microcapsules presented by de Souza and Al-Tabbaa (2018) [37] were considered. Two different microcapsules, each with different shell properties, were considered, denoted BH, which had a colloidal silica core, and BI, which had a mineral oil core. The microcapsule properties and both experimental and model predictions can be seen in Table 2. In the simulations, a perfect bond was assumed.

It can be seen from Table 2 that the numerical predictions are in good agreement with the experimental observations, noting that the simulations assumed a perfect bond and that when the experimental cases did not rupture, this was attributed to a poor interfacial bond [37].

**Figure 6:**
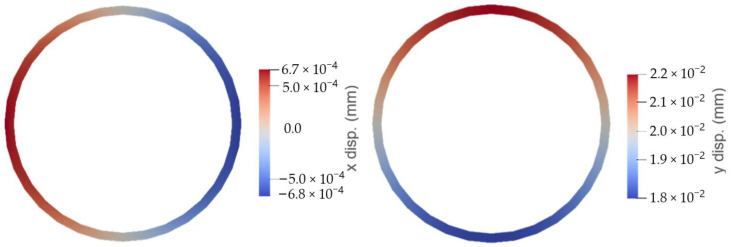
Numerical predictions of displacements in microcapsules.

Typical results of the predicted displacements in the microcapsule (shown here for the BH capsule at fracture) can be seen in Figure 6.

For the virtual testing of physical triggering, the microcapsules considered, chosen based on the results presented in Section 3.1 and Section 3.2, as well as experimental reports, were 600 μm in diameter with a shell thickness of 50 μm. A cementitious matrix material with the following properties, a Young’s modulus of E = 30 GPa, a tensile strength of f_ctm_ = 3 MPa and a fracture energy of G_f_ = 0.1 N/mm, was employed. A range of relative values for both the interfacial transition zone and the microcapsules, again chosen based on the results presented in Section 3.1 and Section 3.2, as well as experimental reports, were investigated, as detailed in Table 3. The relative terms were calculated as the ratio of the parameter divided by the corresponding value for the cementitious matrix. For example, relative capsule stiffness is given as the capsule stiffness divided by the cementitious matrix stiffness. The use of relative values in the presentation of the results and as the input parameters for the machine learning ensures generality, as it is the mismatch in properties between the capsules and the matrix that determines fracture response (i.e., fracture of microcapsules vs. fracture strain) [15]. In total, 20,412 different cases were considered. To simulate such a large number of cases, a batch script was written to both create input files and run the program for each case.

**Figure 7:**
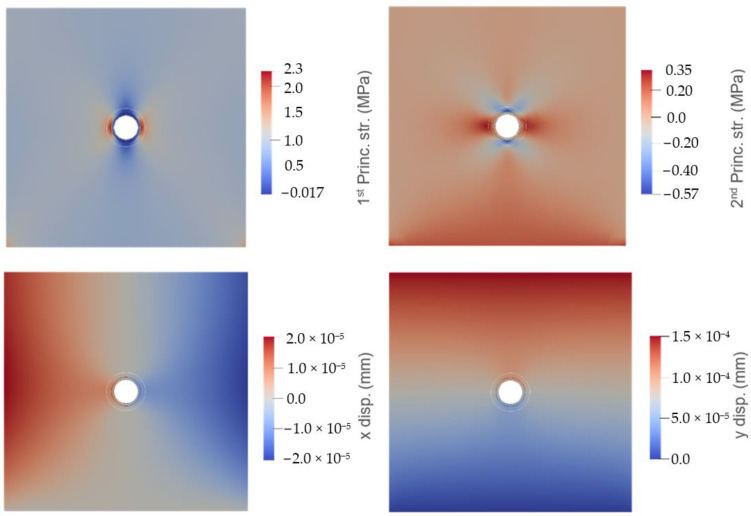
Typical contour plots of results showing: (**top left**) first principal stress; (**top right**) second principal stress; (**bottom left**) displacement in x; and (**bottom right**) displacement in y.

Typical contours of the principal stresses and displacements predicted from the analyses can be seen in Figure 7. The figure shows that, in this case, the major principal stress is concentrated in the cementitious matrix just outside of the interfacial transition zone. This is because the stress concentrates around the circular inclusion, and in this case, the capsule was considered softer than the matrix material and, as such, was under less stress at the same level of strain.

The main output of the code was a data file that recorded the material properties for each case, along with a binary indication of whether fracture occurred (represented with a 1 and a 0, respectively). Using these results, fracture maps could be created to indicate whether or not physical triggering of the microcapsules could be expected for given combinations of material properties. In the present work, however, these results were used to train a machine learning model that can be used as a tool to predict physical triggering for given mechanical properties of the matrix, interface, and microcapsule shell. In addition, using the machine learning model to predict intermediate cases, fracture maps can be created with a greater resolution. In this sense, the validated finite element model was used as a data generator for machine learning.

### 3.4. Machine Learning

The data used for training—as seen in Table 3—were chosen based on experimental reports, being split by 90–10% for training and validation, respectively. The network architecture as well as the hyperparameters are shown in the Table 4.

There is no accepted rule for the choice of the number of hidden layers or the number of neurons within each layer [28], and so the choice was based on a suitable reduction in loss combined with hyperparameter tuning. Similarly, the choice of the ReLU activation function was due to its ability to offer a more generalised fit to the underlying data [41], as well as mitigating the vanishing gradient problem [42]. Here, we use the cross-entropy loss function to assess the training performance of the network, and in doing so, we must apply a softmax filter to render the output in terms of the class probability. In this way, as the probability of fracture or non-fracture approaches 1, the loss will tend to be 0.

**Figure 8:**
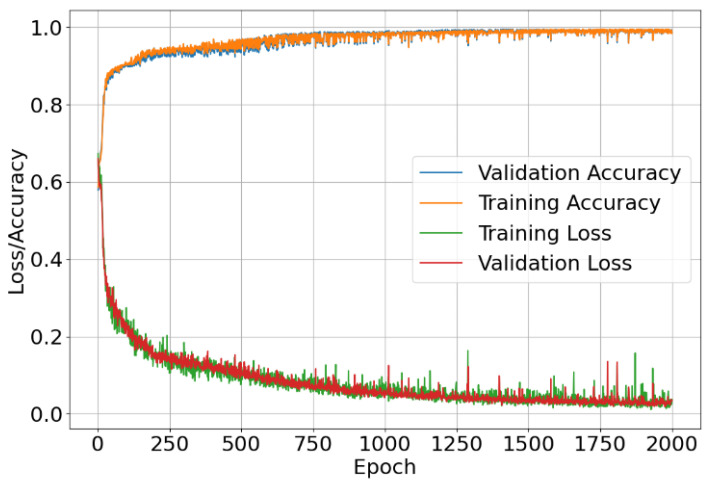
Epoch versus accuracy and loss plots on training and validation data.

Figure 8 shows the learning curves of both accuracy and loss in and out of the sample when training the network. It can be seen that as the number of epochs increases, the loss and accuracy both decrease and increase, respectively, indicating a successful training program [43]. However, we do see a number of larger spikes in the loss. This can be attributed to the optimisation of the network through stochastic gradient descent, along with the choice of the cross-entropy loss function. Increasing the number of epochs to greater than 2000 leads to plateauing of the learning curves and subsequent deviations in loss from the desired trend, suggesting that 2000 was a suitable choice for maximal training cycles.

What is clear is that the learning curves show a good fit to the dataset, as both training and validation curves for loss and accuracy remain close with little variation between them. During training, it was seen that the loss was sensitive to the batch size and learning rate. Taking larger batch sizes reduced the variance in the loss, and a similar response was seen when reducing the learning rate and increasing the number of epochs. From Figure 8, we can determine that the ANN model can successfully predict the fracture behaviour of microcapsules to a sufficient degree of accuracy [44].

**Figure 9:**
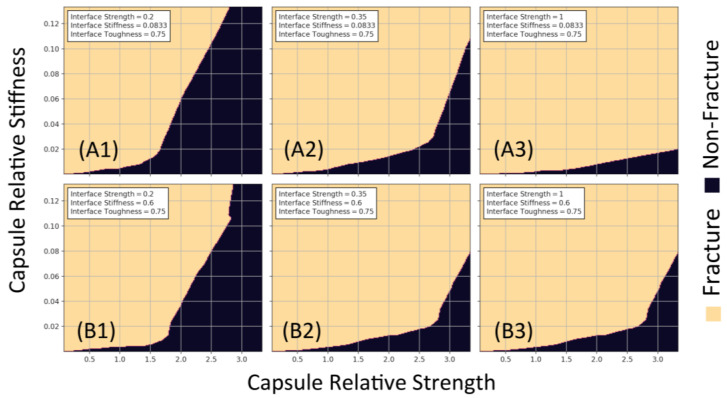
Design curves for the relationship between relative capsule strength and stiffness. The relative interfacial toughness is 0.75: row (**A1**–**A3**) and row (**B1**–**B3**) have relative interfacial stiffness of 0.08333 and 0.6, respectively. Finally, the values of relative interfacial strength are as follows: (**A1**/**B1**) 0.2, (**A2**/**B2**) 0.35, and (**A3**/**B3**) 1.

**Figure 10:**
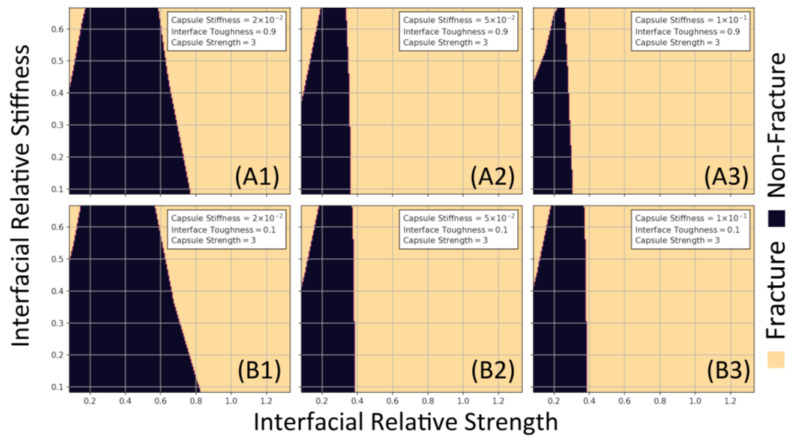
Design curves for the relationship between relative interfacial strength and stiffness. The relative capsule strength is 3: row (**A1**–**A3**) and row (**B1**–**B3**) have relative interfacial toughness of 0.9 and 0.1, respectively. Finally, the values of relative interfacial strength are as follows: (**A1**/**B1**) 2 × 10^−2^, (**A2**/**B2**) 5 × 10^−2^, and (**A3**/**B3**) 1 × 10^−1^.

The trained model was then used to determine design curves to assess conditions for fracture and non-fracture [45]. Parameter values were chosen from the ranges given in the dataset such that both responses (fracture and non-fracture) were observed as opposed to a homogenised response. Figure 9 portrays the relation between relative capsule stiffness and strength where the interfacial toughness is constant for all curves: rows A and B have differing interfacial stiffness, and each column varies with interfacial strength increasing from left to right. By increasing the stiffness of the interface, the model predicts an increased likelihood of fracture as seen by the difference in fracture area of row A and row B in Figure 9. The rise in likelihood is much more pronounced when considering increasing the interfacial strength, as seen in A1–A3 and B1–B3 of Figure 10.

Similarly, the relationship between interfacial stiffness and strength is presented (see Figure 10). Here, the capsule strength is constant for all curves, the interfacial toughness varies from rows A to B, and the capsule stiffness increases from left to right in each column. The same relations observed in Figure 9 can also be seen in Figure 10, such that increasing capsule stiffness results in a greater chance of fracture, with the same holding true for increasing interface strength.

**Figure 11:**
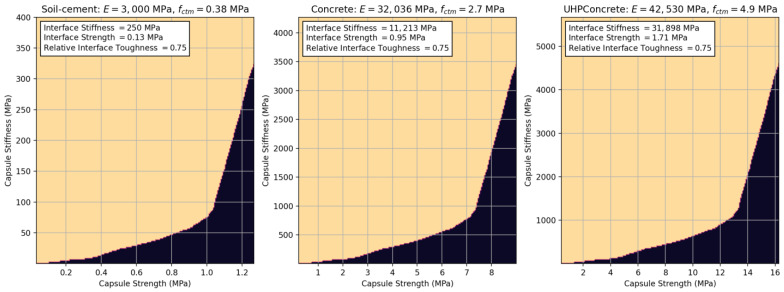
Predicted design curves applied to soil–cement, concrete and ultra-high-performance concrete.

The curves are presented as an effective way to assess capsule response in the design phase, reducing the need to test many cases experimentally, speeding up the process and reducing the resources needed. To highlight how this can be achieved, the relationships observed between relative capsule stiffness and strength were applied to soil–cement, concrete, and ultra-high-performance concrete (see Figure 11). To scale the relationships, Young’s modulus and the tensile strength of all cases were found either directly in the literature [46] or calculated in accordance with Eurocode EN 1992-1-1 [47,48,49]. The calculation of the design curves in Figure 11 highlights the flexibility of the given relative relationships in facilitating the design of capsule characteristics that can lead to physical triggering for a given material.

## 4. Conclusions

This study demonstrates a robust data-driven approach combining numerical modelling and machine learning to tailor microcapsule properties for cementitious self-healing applications. The continuum damage mechanics model enabled virtual testing of a large parametric space to generate a rich dataset relating capsule properties to triggering behaviour in the heterogeneous cementitious matrix. The model was successful in capturing the complex interactions between the capsule shell, interfacial transition zone, and evolving matrix properties. An artificial neural network effectively leveraged the simulation data to predict microcapsule fracturing with high accuracy. The trained model produced design curves that link key parameters, including shell thickness, strength, and interfacial bond strength. This reduces the need for extensive physical testing of capsules. However, targeted experiments are still required to validate model predictions before industrial implementation. Overall, the integrated modelling and machine learning framework shows promise for efficient optimisation of microcapsules tailored for triggering within the complex evolving microstructure of concrete. Further work should focus on quantifying the local mechanical properties more accurately as model inputs and expanding the approach to optimise the distribution and volume fractions of self-healing components. This could move the methodology towards the holistic design of robust and durable self-healing cementitious materials.

## Figures and Tables

**Table 1:** Input and output of network.

	Variables	Description	Range
Input	Capsule	Relative strength	0.08333–3.33333
		Relative stiffness	1.3 × 10^−5^–0.13333
	Interface	Relative strength	0.08333–1
		Relative stiffness	0.08333–0.66667
		Relative toughness	0.01–1
Output		Fracture	1
		Non-fracture	0

**Table 2:** Microcapsule properties and whether fracture occurred.

Capsule	E, GPa	F_ctm_, MPa	Exp. Fracture *	Num. Fracture
BH	2.6	35.2	1/3	1/1
BI	2.4	14.8	2/3	1/1

* The number of cases for which fracture occurred out of number considered; note, the number of cases in experiment considered due to variation in properties.

**Table 3:** Range of relative values considered.

Variables	Description	Range
Capsule	Relative strength	0.08333–3.33333
	Relative stiffness	1.3 × 10^−5^–0.13333
Interface	Relative strength	0.08333–1
	Relative stiffness	0.08333–0.66667
	Relative toughness	0.01–1

**Table 4:** Network architecture and hyperparameters.

Parameters	Values	Description
Activation function 1	ReLU	Rectified linear activation unit
Activation function 2	ReLU	
Loss function	CELoss	Cross-entropy loss
Batch size	5000	
Learning rate	0.001	
Epochs	2000	
Number of hidden layers	2	
Number of neurons in hidden layers	25	

## Data Availability

Information on the data underpinning the results presented here, including how to access them, can be found in the Cardiff University data catalogue at (http://doi.org/10.17035/d.2023.0300392743). Similarly, the trained machine learning model utilised in the paper can be found at https://github.com/EJRicketts/CapNet.
